# Addressing the second victim phenomenon in Israeli health care institutions

**DOI:** 10.1186/s13584-023-00578-5

**Published:** 2023-09-04

**Authors:** Rinat Cohen, Yael Sela, Rachel Nissanholtz-Gannot

**Affiliations:** 1 Department of Health System Management, School of Health Science, Ariel University, Ariel, Israel; 2 Nursing Department, Ruppin Academic College, Emek-Hefer, Israel; 3 Smokler Center for Health Policy Research, Meyers JDC-Brookdale Institute, Jerusalem, Israel; 4 Nursing Department, Ramat Gan Academic College, Ramat Gan, Israel; 5 Rishon Le Zion, Israel

**Keywords:** Risk management, Patient safety, Second victim, Adverse events, Healthcare policy

## Abstract

**Background:**

The ‘second victim’ phenomenon (SVP) refers to practitioners who experience a negative physical or emotional response, as well as a professional decline, after participating or witnessing an adverse event. Despite the Israeli Ministry of Health’s implementation of specific protocols regarding the overall management of adverse events in health organizations over the past decade, there is limited knowledge regarding healthcare managers’ perceptions of the ‘second victim’ occurrence.

**Methods:**

A phenomenological qualitative approach was used to identify an accurate view of policy. Fifteen senior risk manager/and policy makers were interviewed about their knowledge and perceptions of the ‘second victim’. Topics addressed included reporting mechanisms of an adverse event, the degree of organizational awareness of ‘second victim’, and identifying components of possible intervention programs and challenges to implementing those programs.

**Results:**

Examining current procedures reveals that there is limited knowledge about uniform guidance for health care organizations on how to identify, treat, or prevent SVP among providers. The employee support programs that were offered were sporadic in nature and depended on the initiative of a direct manager or the risk manager.

**Conclusions:**

Currently, there is little information or organizational discussion about the possible negative effects of AE on healthcare practitioners. To provide overall medical care that is safe and effective for patients, the health system must also provide a suitable response to the needs of the medical provider. This could be achieved by establishing a national policy for all healthcare organizations to follow, raising awareness of the possible occurrence of SVP, and creating a standard for the subsequent identification, treatment and future prevention for providers who may be suffering.

## Introduction

An adverse event (AE) in healthcare may be caused by medical error, patient endangerment or patient trauma and may have additional negative effects on the provider’s emotional and physical health, and future professional functioning [1]. Since Wu’s initial supposition [2] that an AE can have three victims, the patient as the main "first victim", the medical provider as “second victim” and the organization itself as “third victim” [3], the definition of the second victim phenomenon (SVP) has been expanded to include a provider’s emotional response to any negative event related to patient care, even if no error were made or harm caused to the first victim [3–5]. In 2022, an international group of experts finalized a consensus definition of the second victim as "Any health care worker, directly or indirectly involved in an unanticipated adverse patient event, unintentional healthcare error, or patient injury, and becomes victimized in the sense that also the worker is negatively impacted” [6, p. 6].

Providers experiencing SVP express difficulty coping with an overflow of negative emotions that may appear immediately after an AE or, alternatively, years later [5–9]. Sleep disorders, eating disorders, concentration and memory disorders, alcohol and drug use, anxiety, post-traumatic stress disorder (PTSD), and even suicide attempts, have been associated with SVP as well [10–14]. Organizational support provided immediately following the event may help nurses and physicians recover more quickly and return to their previous level of professional functioning [15]. On the other hand, ignoring the unique needs of the ‘second victim’ may delay or prevent recovery, and lead to the provision of defensive or suboptimal treatment [15–19], since providers may doubt their clinical skills and professionalism [20–22] and consider leaving, or do leave, the profession [5, 10, 16, 22].

Therefore, some institutions have developed intervention programs [23], often based on the guidelines of the United States Agency of Healthcare Research and Quality (AHRQ), and include organizational mechanisms for implementing policies and raising awareness of the phenomenon. Additionally, these programs often include more proactive measures such as: identifying staff members at risk for SVP, training dedicated staff members to offer guidance, and providing immediate and long-term support as needed [24].

A recent systematic review of support services offered to nurses synthesized results from intervention programs developed among ten institutions [23]. In the majority of the programs, the availability of support did not depend on reporting an AE. When support was offered, it was provided by employees who had received organizational training; all conversations were confidential, and further therapeutic support was often funded by the organization. Such programs have been shown to improve personal and organizational functioning, as well as cost–benefit indices [23, 25, 26]. Yet, despite the advantages of such programs, many health systems around the world do not yet incorporate support in a manner that adequately meet the needs of ‘second victims’ [7, 9, 23, 27–29].

In 2006, the first regulatory protocol in Israel was published by the Ministry of Health (MOH) to manage the process of an AE management and reduce the risk of future events [30]. Over time, the MOH has strengthened this process to guide health organizations on how to identify, report, manage, treat and hopefully prevent, future AEs. There is a clear policy that includes numerous references to the patient (first victim) and legal consequences for the organization (third victim) [31–34]. However, there is little reference to the possibility of the treating staff being harmed themselves by the AE. Where the practitioner is mentioned, it is written that it is necessary to support practitioners involved in an AE, but there is no reference to the possibility of the practitioner being a "second victim, nor acknowledgement of SVP or support provided to the provider. Moreover, there is no current regulation that requires assessment, assistance or support offered to the provider after such an event [32, 33].

In Israel, there have been limited studies conducted on the SVP. An analysis of 150 nurses’ interactions with suicidal patients, showed that, in many cases, nurses’ responses led to SVP symptoms and could have contributed to nurse absenteeism and turnover, even years after the event [7]. One other study compared nurses’ responses to making a medication error at two points in time (2005 compared with 2018) and found that when the organizational risk management team took a non-blameful approach to errors, more positive second-victim functioning was found [35]. The only larger scale review thus far that has been conducted on SVP in Israel, suggested that organizations create an organized system to manage the effects, and not respond, ad hoc, in the moment of crisis [36]. The summarizing points of these studies demonstrate a need for healthcare organizations to recognize the impact of SVP and provide appropriate support to affected providers.

Therefore, the objectives of this study were to map existing policies in organizations and examine the perceptions and attitudes of risk managers and policy makers regarding SVP in Israel.

## Methods

We used a phenomenological qualitative approach to identify an accurate current view of policy. Upon receipt of the ethics committee approval of the participating academic institution (#AU-20220409) three key leaders within the field of risk management research in healthcare systems were identified and contacted. To generate a wider perspective, these key leaders then identified upper-level administrators within the following healthcare areas: MOH administration, health funds, hospitals, long -term hospitalization facilities, and community health services. Of the 18 individuals subsequently approached to participate, first via email and then by phone, 15 consented to be interviewed.

### Research process

Semi-structured, open-ended interviews were conducted, via Zoom (Zoom Video Communications software), between June and December 2022. At the beginning of the interview, the purpose of the study was explained, and all interviewees signed an informed consent form. Interviews lasted between 60 and 90 min and were assisted by a previously developed interview guide [37]. Interviews were recorded in accordance with the interviewee's consent. The data collection process was an iterative process that included collecting, coding, and analyzing data [38]. Sample size was based on data saturation until no new information was obtained from additional participants [39]. To increase the reliability of the results, the interviewer summarized each interview to the interviewee at the end in order to allow the participant to check for and clarify any misconceptions or add additional information [40]. At no point were identifying details revealed. The decision on the anonymity of the interviewees in the study stems from the fact that the interviewees occupy senior positions within the health system, and there was concern that interviewees would refrain from critiquing or expressing disagreement with organizational policies if the interviews were not anonymous.

### Interview guide

Interview questions were based on similar studies [5, 23, 26] and included topics such as: existing reporting mechanisms when an AE is reported/discovered [26]; degree of organizational SVP awareness in the health care system in general in Israel; identifying and mapping factors that promote and hinder the development of support programs [23]; identifying and supporting medical staff who express second victim characteristics [5]; optimal ways to develop relevant clinical and professional training [26].

### Data analysis

Interviews were analyzed using thematic analysis [40] with the following steps: 1. Interview transcription: 2. Multiple reading iterations to create a list of themes: 3. Breaking down the text into building blocks for further analysis: 4. Open coding of the material, with the aim of creating categories that represented emerging themes: 5. Extraction of the main categories that have formed into themes 6. Identifying connections and relationships among the different themes. Data analysis ended when all categories were saturated and the relationships between them were based on significant data, integrated into a story or description [41]. Analyzing the data in this way made it possible to examine the perceptions of the interviewees more deeply regarding the phenomenon of the second victim, and their position regarding the development of support programs.

## Results

### Socio-demographic findings

To provide adequate representation, the study population included 15 participants with relevant roles in risk management and quality assurance in health funds (3 participants), hospital-based systems (2), the Israeli MOH (4), long- term hospitalization units (3), and researchers and lecturers who teach quality assurance and/or risk management in nursing schools (3). All interviewees had worked in clinical field positions for at least an average of 15 years and held general management positions in risk management and/or quality assurance (Table 1). In addition, eight of the 15 interviewees were academic lecturers as well. The average age of the participants was 45 (40–65).

**Table 1:** Sociodemographic characteristics of the interviewees

Variables	Type	Amount
Gender	Men	3
	Women	12
Age	Mean = 45 (40–65 year)	
Education level	MD, PhD, Associate Professor	2
	RN, PhD	3
	RN, MA	10
Professional position	Head Education Authority Associate Dean-School of Medicine	2
	Researchers and lecturers who teach quality assurance and risk management in nursing schools	3
	Representatives of regulatory bodies	4
	Director of patient safety (MOH)	
	Area director for public health/ community-based care system (MOH)	
	Supervisor of chronic hospitalization (MOH)	
	Director of nursing competency (MOH)	
	Risk management directors for 3 of the 4 health funds	3
	Head nurses at long term hospitalization facilities	3
Professional seniority in risk management	Mean = 7 (2–10 years)	
Professional organization	University	3
	Medical Centers	2
	Ministry of Health	4
	Long term hospitalization facilities	3
	Health fund	3

Only five participants dealt with risk management and quality assurance exclusively. The rest were engaged in the field of risk management as an additional responsibility within their position, which theoretically constituted between 15 and 20% of their overall positional responsibilities.

The four main themes identified after data saturation were: (1) Senior manager perceptions regarding the definition of SVP, actual risk factors of the problem, including the scope and potential consequences of SVP; (2) The presence of organizational support programs for medical staff experiencing an AE; (3) Aspects of existing support programs for providers; and (4) Challenges in developing relevant future support policies (Table 2).

**Table 2:** Summary of categories and main themes

Category	Main themes
Perceptions of managers and policy makers, regarding the SVP—definition, risk factors scope, and potential consequences	*Perception of definition*: The risk of suffering from the SVP is connected to the severity or actual damage caused to the patient
	*Perception of risk factors*: 1. Professional seniority- younger staff are at higher risk 2. Intensity of the work environment- extreme events, and acute clinical settings increase the risk of SVP 3. Awareness of the phenomenon- most practitioners in various sector do not know the phenomenon and do not seek help
	*Perception of scope and potential consequences:* 1. The phenomenon is considered marginal related to an error and/or actual harm to the patient 2. Most practitioners understand how to separate personal experience and adequate treatment and return to complete functioning 3. There is no connection between burnout, dropout and SVP 4. There is no connection between risk management and dropout rates in an organization
Presence of organizational support programs following AE	A main goal of a risk management unit is to identify AE, map processes with risk potential, draw conclusions and conduct organizational learning. A risk management system in every institution is structured and organized
	There is no operational policy to identify or provide emotional support to a practitioner who has been involved in an AE
	Addressing the emotional needs of the practitioner depends greatly on the individual managing the event, and the manager’s awareness of SVP
	As of 2021, under the auspices of directed support for programs that improve safety culture, budgetary resources have been allocated without specified content or scope of the training
	In several organizations, there are local /sporadic programs, mainly during crises
	There is no dedicated position to offer supportive treatment to the practitioner [after an AE], nor is there specific training on the topic
Components of developing a support program	The organization has a responsibility to the practitioner; thus, it is important to develop a support program for the ‘second victim’
	There is a disagreement between operating an anonymous hotline versus training colleagues or direct managers, as an initial response
	There is a difference of opinion as to whether it is recommended to take a proactive organizational approach and offer assistance from the onset, or take a passive position and provide help when the practitioner requests support
	There is a difference of opinion as to whether initiating a support program should be dependent on the submission of an adverse event report
	Appropriate training for peer support / risk managers
	Separation between managerial and treatment roles
Challenges in developing support programs	Allocated budget
	Suitable staff
	Compliance—stigma and labeling of ‘second victim’ as a barrier to identify and treat practitioners
	Organizational culture that creates feelings of fear and anxiety about losing anonymity and livelihood

## Perceptions regarding the phenomenon of the second victim: definition, risk factors, scope, and possible consequences

### Definition

One senior manager stated that she was familiar with the definition of SVP [5–3] and understood the phenomenon in depth as she deals with it in her research, and therefore works to raise awareness and develop an appropriate response within her organization.

I know the phenomenon in depth and have been researching it for the past few years. In my organization, we have established a dedicated team that deals with the "second victim" phenomenon in a routine manner that operates for the benefit of all staff members in the organization.

Apart from her, the rest of the interviewees stated that they had only superficial familiarity with the concept of second victim, a familiarity based on general knowledge and previous experience, but not based on specific training. Moreover, ten interviewees stated that, in their opinion, suffering from the SVP was related to making a serious error in treatment, or when significant harm (such as serious injury or death) was caused to the patient.

I've heard the term here and there in seminars. Although I understand that after an adverse event, there can be a harmful effect on the practitioner, if a serious mistake was made or significant damage was caused, but we usually talk less about it or deal with it in our daily lives.

This is the first time I have heard this as a professional definition, now, from you. I know the phenomenon, but I don’t deal with it; it’s not related to my job.

### Risk factors

Twelve of the interviewees referred to professional seniority as a protective factor and believed that younger practitioners are at higher risk of experiencing the phenomenon than more experienced medical staff.

It is logical that more senior staff would be more skilled and have more inner resilience to cope with these events, than younger staff.

Another risk factor that was emphasized was the intensity of the work environment, where eight of the interviewees stated that it is likely that the phenomenon is more prevalent in intense work environments, where providers meet with serious and complex clinical situations on a regular basis and must cope with immediate life and death encounters.

I think that when workers are stressed and dealing with constant life or death issues, and the level of stress is much higher and they make a serious error, that could truly influence the provider.

According to six of the interviewees, most of the practitioners themselves were not familiar with the phenomenon or aware of possible consequences and had not received any training on the subject during their professional career.

The very fact that the phenomenon is not recognized by the practitioner can cause the practitioner to not seek help. Nurses [and doctors] will not recognize the problem and not seek help if they experience such a response to an AE. This taxing solo journey alone increases the risks of SVP.

From the three interviews with the lecturers who teach quality assurance and risk management in nursing schools, it seems that risk management is taught on a broad scale and mainly refers to the management of an AE: prevention, reporting, and legal management. However, little time is devoted to the SVP at all (approximately 1–2 h within the four-year degree program).

As part of the bachelor's degree in nursing, students are exposed to the SVP during a class or several classes in connection with AE. However, I can’t guarantee if they understand what the phenomenon means. Students are mainly taught the principles of identifying and writing an incident report.

First, they learn "First, do no harm" and talk a lot about our role in keeping the patient safe. They also touch a little on the consequences on the nurse if a patient is harmed, but I don’t know if they remember from all the material they learned, specifically to put a finger on the SVP. Of course, they teach about emotional and behavioral consequences for nurses when exposed to traumatic events. Is this understood in depth? I can’t tell you. The basic premise is that a nursing student will get tools or improve skills with various issues in the field. It is expected from the system itself, from in-service education and initial on-the-job training, to teach the nurse and give specific tools for those cases.

### Scope of SVP and potential consequences

Ten of the 15 interviewees estimated that the phenomenon is marginal and not widespread within the organization. Moreover, they believed it was related to a significant error and/or actual harm caused to the patient and truly only affected the primary care team or those that had direct interaction with the patient. Most of the interviewees stated that even after such a serious event, the practitioner may experience difficult emotions such as shock, shame and guilt. However, after a short period, even then the practitioner will recover and return to full function.

It is part of our dynamic day-to-day work, and the people in the field know how to deal with it.

The majority of the interviewees (12/15) did not refer to possible long-term effects on the mental or physical health of the practitioner. Twelve interviewees believed that the practitioners were able to make a "separation" between difficult emotions and their professional functioning so that they could continue to work and provide quality and adequate care in any situation.

I trust our providers know how to separate and cope, and if something is wrong, they know how to seek help.

The vast majority (13/15) did not mention possible professional repercussions, such as attrition, burnout, practicing defensive medicine, etc.

I do not see a connection between dropping out and the SVP. Most employees leave the system for reasons of convenience; that’s how it is with the new generation.

We support the practitioner all the way from the moment the mistake is discovered, but regarding disciplinary action, we will take a step back. That is, the role of human resources, there should be no connection between risk management and disciplinary measures.

### Presence of organizational support for the second victim phenomenon

With a single exception, from the interviews the extensive provisions taken for the prevention of AE do not include a set protocol for providing post-event emotional support to practitioners.

I’m researching this issue. We presented it [our findings] to the MOH at one of the conferences, and we even received a dedicated budget for the hospital. Our organization routinely follows AHRQ recommendations - to locate, monitor, and treat providers with SVP, but what happens in other organizations I can’t say. I don’t know of a uniform policy.

Other than this interviewee, the remaining participants did not know of protocols or policies within the Israeli MOH or their organizations specifically offering support to providers after an AE. A risk manager at one of the health insurance funds described the process as follows:

We provide professional support in the writing of the incident report: how to identify an AE, how to draft the report, how to send it and to whom, [etc..] and we have routine field training in this area. After a report of an AE is received, the unit team gathers to discuss the incident, and, depending on the nature and severity of the event, an investigation is conducted with the practitioner and the direct supervisor. If necessary, a report is filed with the MOH according to the protocol. Of course, after the investigative process, there is also organizational learning. We do not have guidelines for addressing the emotional or mental needs of the provider. Addressing these needs depends a great deal on the individual conducting the investigation and the degree of the particular risk manager's sensitivity and awareness of the phenomenon.

In 9 of the 15 organizations, no proactive measures were taken to raise awareness of the phenomenon and its consequences among the employees and direct managers in the field.

“In our organization, we do not use the term ‘second victim’.”

The interviewees also stated that they were not familiar with external or internal organizational policies or procedures that provided criteria for how and in what manner medical staff facing SVP should be identified and then provided support. Regarding future plans, four of the interviewees stated that beginning in 2021, as part of the MOH’s budgeted support for improving safety culture, health funds were offered resources with which to develop plans for a safe organizational culture. However, the content and scope of the trainings, nor specific line items, were defined for this general budget. Following this, the interviewees stated that in a number of organizations, there were local sporadic programs, mainly during crises (such as the recent COVID-19 pandemic), according to the decision of a direct manager, but that no systematic policy or routine support programs have been implemented.

Eight out of the 15 interviewees stated that, if the provider reports an AE, and directly addresses the direct manager and requests psychological assistance following that event, he or she will be referred to the risk management department, which will refer the provider to a social worker or a mental health nurse within the organization. However, there is no job definition designated for the care of medical staff after an AE, and those employees have not undergone specific training on the issue. Currently, the decision on the specific assistance and manner in which support will be offered depends on the discretion of the immediate manager and/or the risk manager. For example, a head nurse and risk manager at a geriatric medical center stated:

I suppose we all have baggage, and we carry negative emotions. In principle, there is nothing standardized in our approach; it all depends on the direct manager. In the end, everything is based on the personal relationship [between manager and nurse].

In our organization, I don’t think we pay enough attention to this phenomenon. I'm sure the providers have personal experiences and we're not there enough. We do not investigate these experiences. In very exceptional cases, we sometimes do individual interventions, but that isn’t something that exists routinely.

### Components of support programs

Most (13/15) of the interviewees agreed that the organization has a responsibility toward the practitioner affected by an AE and that in the current situation, proper assistance is not provided. Thus, it is important to develop relevant support services. However, there were differences of opinion as to the specific components and processes of those theoretical services. Regarding optimal initial treatment for SVP, six participants suggested the implementation of an anonymous hotline and a follow-up protocol, while seven felt that colleagues or direct supervisors should be trained to provide an initial response. Others supported the position that a provider may not reach out or share with the direct supervisor because of shame, guilt, or embarrassment.

Two main approaches emerged among the interviewees as to who should initiate the support process. One approach considered the junior management level, the direct supervisor, as the key figure to identify an employee suffering from SVP. Five of the interviewees see the direct manager as a key figure for identifying an employee suffering from the phenomenon and that this manager should provide support.

A head nurse is the first to identify SVP and provide an immediate and effective response.

The main reasons for this included personal familiarity with events in the field and with the providers and the ability to respond in real time:

The organization should train the direct manager to be able to support the practitioner. The direct supervisor is close by, knows these practitioners, understands the situation and can provide immediate support to the staff.

They also stated that a supervisor should be trained to manage the issue within the organization, within the risk management department or with continued education programming.

In contrast, interviewees suggested that practitioners be allowed to appeal directly to the person in charge of the issue within the risk management department without the knowledge of the direct supervisor. A main component of a support services program should include proactive steps to increase reporting and guarantee anonymity because, in the opinion of six interviewees, nurses will choose not to report due to feelings of fear, embarrassment and fear of being fired.

The provider may fear a violation of anonymity, or their team members' reactions, and [possible] future consequences for continued employment.

It is not right that a direct manager should also be the therapist. The providers will not be honest for fear that they will be labeled, and this will prevent future promotions.

Ten of the interviewees did not perceive their role as one who should provide direct treatment or support providers, as they felt that they did not have the proper training*.* Suggestions for a possible supporter included a dedicated psychologist, lawyer, mental health nurse, psychiatrist, or organizational consultant.

If necessary, the provider can be referred to the district mental health nurse.

Five participants believed that if there is a report of a serious AE, with severe consequences to the patient, the risk management departments that manage the investigation should include both a structured reference to the provider’s emotional response and provide tailor-made support, if necessary. However, three of the interviewees believed that the support should only be given upon request and that the very initiative of contacting the provider could be embarrassing.

It is very important to give appropriate support to the provider as well. Nevertheless, we do not use the term "victim" in our organization. It is hard to recruit new teams anyway: people who choose to help others should not feel like victims. We need to find another name for the phenomenon. We should support the practitioner without labeling him as a victim.

### Challenges to the development of support services

Thirteen interviewees cited the issue of the lack of an allocated budget, together with possible lack of cooperation on the part of the providers, as two main challenges:

In every decision or policy change in the organization, budget considerations are the main criterion for the decision. It is necessary to train dedicated staff members and create [relevant] job descriptions. All of this requires a budget;therefore, the organization needs to raise the phenomenon to the order of utmost importance and allocate a dedicated budget for the development of support mechanisms for the care teams.

Even if we set up programs of this type, it’s not certain that there will be responsiveness on the part of the practitioners. During the COVID-19 pandemic, several attempts were made to respond to the emotional needs of the teams, but there was very little responsiveness, if at all. Maybe if we offered training to the entire medical team and legitimized the phenomenon, then maybe there would be more cooperation [on the part of the staff].

In addition, 13 interviewees stated that comprehensive training must be provided to all practitioners within the organization, including junior managers, to increase awareness of the existence of SVP. Some of the interviewees stated that it is necessary to build an organizational mechanism for monitoring and assessing the severity of the phenomenon and its scope in the organization and then build structured work processes and protocols for providing assistance to the practitioner, according to the severity of the impact. For such a plan to come to fruition, the organization needs to prioritize the issue as part of a policy to promote quality of care and patient safety and allocate dedicated resources and positions. However, to allocate a budget, the practitioner must first show willingness and cooperation to receive help when needed. In connection with this, six of the fifteen interviewees stated,

In our organization, it is possible to request support from a psychologist, organizational consultant, or mental health nurse, but the medical team’s usage of these services is quite low.

In their opinion, not every employee reacts in the same way to AE's; they mainly react to difficult events. Therefore, it is a mistake to contact everyone involved in an AE, but rather that assistance be provided only to the employee(s) who reported the need for support.

## Discussion

The purpose of a risk management unit is to identify AEs, map processes with risk potential, draw conclusions and implement organizational learning from events that have occurred, all with the aim of improving patient safety and overall quality of care [30–34]. These units also provide guidance and information regarding the legal aspects of AE management. Despite the extensive activity of the healthcare system to learn from AE's and prevent future events, our findings show that Israeli Ministry of Health protocols that guide healthcare organization on how to manage an AE, are focused on the event itself and the prevention of its recurrence and not on the needs of the provider or on support mechanism.

In our study, despite the high level of senior management positions held, most of the interviewees reported that they had only superficial knowledge of SVP, contrary to their knowledge of PTSD. The employee support programs that were offered were sporadic in nature and depended on the initiative of a direct manager or the risk manager. These responses mirrored results from a study that surveyed members of American Society for Healthcare Risk Management about the presence, features, and perceived efficacy of their organization’s provider support programs [28]. The review covered 575 healthcare institutions in the United States and found that the majority (73.6%) of respondents reported that their organization had some form of program in place, yet there was little uniformity among protocols and practices [28]; very few adhered to the recommendations suggested by the American Agency of Healthcare Research and Quality (AHRQ), that recommends that support be offered after an AE in order to prevent development of SVP symptoms [24]. In both our study and the review [28], respondents also reported barriers to developing adequate programming, including budgetary constraints, maintaining suitable support teams and lack of staff compliance.

In our study, the interviewees agreed that organizations shoulder the responsibility if a practitioner’s health and functioning are damaged as a result of AE involvement; they also acknowledged that, in theory, a provider may experience emotional distress if patient harm occurred over their action or inaction. This perspective suggests that these managers may view SVP occurring only in the case of error, which is in contrast to the current definition of the "second victim", where any negative or unexpected experience during care may lead to SVP [3, 5, 6]. In a sense, managers focused on providers’ emotional states only if they had been directly involved in an event where the patient was seriously harmed; this has been seen in other studies as well [7, 10, 42, 43].

Moreover, our data demonstrated that support provided depended mainly on the individual’s own request for help, or after the report of an AE with serious patient consequences. This contrasts with the recommendations of the AHRQ, which suggest the maintenance and delivery of comprehensive support services, irrespective of direct request [24].

Conversely, many health organizations have initiated employee intervention programs based on AHRQ guidelines [23, 25, 28, 44]. An expansive review of 10 intervention programs from the United States, Spain and Indonesia, from 2006 to 2017 found that, in most programs, support was voluntary, available immediately or shortly after the event, and offered through trained colleagues, with further confidential professional support by psychologist, psychiatrist or social worker trained to treat second victim providers offered when needed. This review also mentioned that the more the awareness of SVP increases, the more the responsiveness and uptake of organizationally provided support increases [23]. However, in our study, we identified only one hospital that was operating in accordance with the AHRQ guidelines.

Most of the interviewees believed that the organization's ability to provide support depended, first and foremost, on the provider’s request for formal support. From the point of view of the interviewees, the organization is "passive" in detecting the SVP. However, this statement contradicts the fact that many practitioners report difficulty in asking for help in this matter, due to barriers such as lack of awareness, and emotions such as shame, guilt, fear of the consequences, and fear of exposure [17, 27]. The lack of awareness of the phenomenon and the lack of reporting lead to a delay in receiving the assistance required for the practitioner in all phases of coping [27] as well as the ability to determine accurate estimates of the extent of caregivers experiencing the phenomenon [45].

Finally, in this study, the interviewees were divided regarding the preferred way to identify staff in need and offer subsequent support. Some participants expressed the need to train direct managers to both identify possible medical staff suffering from SVP and then provide initial support, proximal to the actual event. Others felt that there should be a complete separation between the administrative role of the manager and the therapeutic needs of the provider. Recent research does support the need [19, 26] that there be separation between management and proffered psychosocial support, by a non-biased, confidential professional, trained to treat second victims. Health organizations need a referral mechanism to handle this situation that is confidential and respects providers’ confidentiality. Collegial support seems the most desirable [46, 47].

## Conclusion

Given that health organizations in Israel have diverse organizational cultures, and the phenomenon of the second victim in the healthcare system is a broad and cross-sector problem, it is vital to formulate a comprehensible and binding [47, 48] approach that is overarching. A recent Israeli qualitative study [49] found that the majority of nursing staff exposed to an AE need support, but do not request it from the organization, nor do they receive proactive support, possibly due to low awareness of the phenomenon, personal barriers and fear of the organizational response.

Although the MOH has scattered, general requirements for policies and procedures addressing the second victim issue [32, 33], the Ministry does not audit institutions for compliance with their policies and procedures; nor has it established a specific budget framework [50], guidelines or measurement of management processes for identifying and treating the second victim. This paradox possibly contributes to the lack of cohesive policies and procedures within individual organizations. Considering the seriousness of the phenomenon and its consequences, creating a strong national policy accompanied by implementation and measurement programming according to AHRQ guidelines, with delineated budgetary line items, appear to be the correct first steps. These programs should include awareness raising among providers, training some colleagues to identify and support the "second victim", and setting standards of follow up care by special trained professionals as social workers or psychologist.

A strong national policy accompanied by implementation and audited for compliance would place SVP on the public agenda and legitimize appeal for help from the organization. It would also outline national guidelines and principles, encouraging healthcare organizations to set goals, and coordinate activity among other organizations, which all would facilitate consistency in decision-making and act as an umbrella action to allocate resources, while raising awareness.

To overcome possible barriers to providers compliance, it is important that the organization initiate specific training, conduct periodic discussions about their emotional responses to an AE, and implement a nonjudgmental inclusive policy regarding possible negative emotions that may accompany participating or witnessing an AE. The implementation of these active measures may help raise the awareness of providers about the phenomenon as well as in identifying SVP at an earlier stage [23, 25, 48].

## Data Availability

The data presented in this study are available on request from the corresponding author. The data are not publicly available due to privacy restrictions.
